# Myeloid and dendritic cells enhance therapeutics-induced cytokine release syndrome features in humanized BRGSF-HIS preclinical model

**DOI:** 10.3389/fimmu.2024.1357716

**Published:** 2024-02-07

**Authors:** Gaëlle H. Martin, Alexis Gonon, Perrine Martin-Jeantet, Florence Renart-Depontieu, Zuzana Biesova, Anokhi Cifuentes, Arnab Mukherjee, Thomas Thisted, Astrid Doerner, Dean O. Campbell, Ludovic Bourré, Edward H. van der Horst, Amélie Rezza, Kader Thiam

**Affiliations:** ^1^ genOway, Lyon, France; ^2^ Sensei Biotherapeutics Inc., Boston, MA, United States; ^3^ Crown Bioscience Inc., San Diego, CA, United States

**Keywords:** humanized preclinical models, cytokine release syndrome, myeloid cells, BRGSF mice, safety assessment, immunotherapy

## Abstract

**Objectives:**

Despite their efficacy, some immunotherapies have been shown to induce immune-related adverse events, including the potentially life-threatening cytokine release syndrome (CRS), calling for reliable and translational preclinical models to predict potential safety issues and investigate their rescue. Here, we tested the reliability of humanized BRGSF mice for the assessment of therapeutics-induced CRS features in preclinical settings.

**Methods:**

BRGSF mice reconstituted with human umbilical cord blood CD34^+^ cells (BRGSF-CBC) were injected with anti-CD3 antibody (OKT3), anti-CD3/CD19 bispecific T-cell engager Blinatumomab, or VISTA-targeting antibody. Human myeloid and dendritic cells’ contribution was investigated in hFlt3L-boosted BRGSF-CBC mice. OKT3 treatment was also tested in human PBMC-reconstituted BRGSF mice (BRGSF-PBMC). Cytokine release, immune cell distribution, and clinical signs were followed.

**Results:**

OKT3 injection in BRGSF-CBC mice induced hallmark features of CRS, specifically inflammatory cytokines release, modifications of immune cell distribution and activation, body weight loss, and temperature drop. hFlt3L-boosted BRGSF-CBC mice displayed enhanced CRS features, revealing a significant role of myeloid and dendritic cells in this process. Clinical CRS-managing treatment Infliximab efficiently attenuated OKT3-induced toxicity. Comparison of OKT3 treatment’s effect on BRGSF-CBC and BRGSF-PBMC mice showed broadened CRS features in BRGSF-CBC mice. CRS-associated features were also observed in hFlt3L-boosted BRGSF-CBC mice upon treatment with other T-cell or myeloid-targeting compounds.

**Conclusions:**

These data show that BRGSF-CBC mice represent a relevant model for the preclinical assessment of CRS and CRS-managing therapies. They also confirm a significant role of myeloid and dendritic cells in CRS development and exhibit the versatility of this model for therapeutics-induced safety assessment.

## Introduction

Predicting the potential toxicity of therapeutics is of utmost importance in drug discovery. Indeed, some compounds can induce side-effects ranging from organ-specific toxicity to a systemic inflammatory response known as cytokine release syndrome (CRS), characterized by a massive release of cytokines (or cytokine storm) that can lead to multiorgan failure. First described as an adverse effect of the anti-T-cell antibody OKT3 (1), an immunosuppressive treatment for solid organ transplantation, CRS has since been reported after treatment with several antibody-based therapies, such as anti-CD20 Rituximab (2) or anti-PD-1 Nivolumab (3). In addition, it represents the most frequent adverse effect associated with T-cell engaging therapies, including bispecific antibodies and CAR-T cells (4). Modelling CRS in preclinical settings represents a critical challenge, especially with the recent and rapid development of novel therapeutics in immuno-oncology.

Different preclinical models can be used to assess CRS (5), with arguably the most appropriate being immunodeficient mice reconstituted with a human immune system (HIS) (6). These HIS mice can be obtained from reconstitution with human adult peripheral blood mononuclear cells (PBMC) or human umbilical cord blood CD34^+^ cells (CBC). Importantly, the features of CRS and timing of its induction is suggested to be compound-dependent (4), adding a level of complexity to its preclinical assessment. Indeed, preclinical models are often tested for CRS assessment following OKT3 treatment, inducing cytokine release as early as 2h and T cell depletion starting 6h post injection, but these features could differ and appear at different time points upon treatment with a different compound. Moreover, PBMC-HIS mice were previously shown to be responsive to CRS-inducing OKT3 treatment, whereas CBC-HIS mice appeared not or less responsive (7).

In order to investigate CRS-like traits triggered by different classes of biologics, and assess the potential contribution of myeloid and dendritic cells in this process, we use here BRGSF mice reconstituted with CBC (thereafter called BRGSF-CBC). BRGSF-CBC mice show systematic and persistent presence of plasmacytoid dendritic cells (pDCs), conventional dendritic cells (cDCs), monocytes/macrophages, and neutrophils, and their myeloid and dendritic compartments can be boosted by hFlt3L treatment (8). In this boosted model, monocytes/macrophages, pDCs, and cDCs’ absolute and relative numbers are increased in the bone marrow and spleen, but also systemically. Importantly, these mice are invalidated for mouse Flk2 (9) which prevents mouse immune cells to respond to hFlt3L treatment, as it is known to cross-react with mouse Flk2. The impact of hFlt3L treatment on immune cell distribution is thus due solely to its binding to human Flk2, expressed by human mature dendritic cells and progenitors, and can act through direct boost of human dendritic cells proliferation and/or human myeloid/dendritic progenitors’ differentiation into myeloid and dendritic cells (10–12). We tested this model’s response to known CRS-inducing compounds treatment, and the involvement of myeloid and dendritic cells in this process. We show that, upon OKT3 treatment, BRGSF-CBC mice exhibited a serum cytokines profile, clinical signs and immune cell distribution associated with CRS, and that boosting myeloid and dendritic cells enhanced these features. We also show that OKT3-induced CRS in BRGSF-CBC mice could be attenuated by anti-TNF-α (Infliximab) treatment (4). When compared to PBMC-reconstituted BRGSF mice (thereafter called BRGSF-PBMC), OKT3 treatment in BRGSF-CBC mice induced a broader range of CRS features, namely a wider spectrum of myeloid-associated cytokines and depletion of cDCs. Finally, we further determined that BRGSF-CBC mice developed CRS-like traits upon injection of Blinatumomab, a bispecific T-cell engager targeting CD3 and CD19 (13), or myeloid-targeting anti-hVISTA (V-domain Ig Suppressor of T-cell Activation) antibody JNJ (14), both known to induce CRS in patients (15, 16). Taken together, these data show that myeloid and dendritic cells enhance CRS-features in BRGSF-CBC mice. They also demonstrate that these mice represent a valuable model for the assessment of therapeutics-induced CRS, and potential CRS management treatments in preclinical settings.

## Methods

### Human samples

Human umbilical cord blood CD34^+^ progenitor cells (CBC; purity >90%) isolated by positive immunomagnetic selection were purchased from Lonza (USA) and CTI Biotech (France). PBMC from healthy donors were purchased from EFS (France).

### Animals

BRGSF mice (BALB/c Rag2^tm1Fwa^ IL2Rγ_c_^tm1Cgn^ SIRPα^NOD^ Flk2^tm1Irl^) were used in this study (genOway, Lyon, France). Mice humanized for the immune system (HIS) were produced following a standardized procedure (17). For BRGSF-CBC mice, newborn females were transplanted intra-hepatically with 7x10^4^ viable human CBC, 24h after full body irradiation conditioning (2.8 Gy; X-ray source). Twelve weeks post-injection, humanization rate and main immune cell proportions were evaluated by flow cytometry in 100µL of peripheral blood, obtained via facial or retro-orbital vein puncture. Flow cytometry analysis of blood cells was performed after leucocyte purification on a Ficoll based gradient (Eurobio Scientific, CMSMSL01-01), and in presence of counting beads. For BRGSF-PBMC mice, 10^7^ viable human PBMC were injected intravenously in 12-weeks old BRGSF females. Animals above 30% of humanization (percentage of human CD45^+^ cells in total number of human and mouse CD45^+^ cells) were included in the experiments. For all experiments, mice were randomized within groups according to donor and humanization rate. Complete immune profiling of mice enrolled in experiments, as well as randomized groups, are provided in **Supplementary Tables 1**–**5** and **Supplementary Figures 1A–C**. Animals were housed in ventilated cages with EOPS sanitary status (19-23˚C, 30-70% humidity, 12-hour night/day cycle), and provided with *ad libitum* access to sterile water and autoclaved feed (SAFE^®^). The experiments involving the use of human cells for the generation of humanized mice were approved by an ethical committee (VetAgro Sup n˚018) and validated by the French Ministry of Education and Research (APAFIS#30015). All other animal experimental procedures were ethically approved by the ethical committee VetAgro Sup n°018, validated by the French Ministry of Education and Research (APAFiS #38721) and performed in line with relevant guidelines and regulations including the EU Directive 2010/63/EU, the related French décret n° 2013-118, and the 3Rs (Refinement, Reduction, Replacement).

### Animal treatments

At 21-22 weeks of age, BRGSF-CBC mice received four intra-peritoneal injections every two to three days (D0, D2, D4 and D7) of 10 μg (in 150 μL of PBS 1X) recombinant human Flt3L (recombinant Flt-3L-Ig (hum/hum), BioXcell, cat. BE0098). Mice were injected by intravenous route with anti-CD3 monoclonal antibody (Orthoclone OKT3^®^; InVivoMAb, BioXcell, BX-BE001-2; 2 mg/kg), Blinatumomab (BLINCYTO^®^ Amgen^®^; 2 or 5 µg/kg), JNJ (Onvatilimab; variable region of JNJ-61610588, now CI-8993, cloned onto human IgG1 backbone, obtained from Sensei Biotherapeutics; 2 or 20 mg/kg), or mIgG2a (Ultra-LEAF™ Purified Mouse IgG2a, κ isotype Ctrl Antibody, Biolegend; 2 mg/kg). Infliximab (INFLECTRA^®^, Pfizer; 2 mg/kg) or hIgG1 (Ultra-LEAF™ Purified Human IgG1 isotype Control Recombinant Antibody, BioLegend; 2 mg/kg) were injected one-hour prior OKT3 treatment. Blood was sampled at indicated time points, mice were sacrificed 24h or 48h post injection, and spleens were collected.

### Clinical monitoring

Body weight and temperature were measured on vigil mice at indicated time points following randomization and initiation of treatment. Temperature was monitored using a Bioseb animal thermometer and a rectal temperature probe for mice.

### Blood sampling and serum preparation

For each time point, at least 150 μL of whole blood was sampled per mouse through the jugular vein under gaseous anesthesia (isoflurane). Whole blood was kept at room temperature for one hour to allow coagulation, then centrifuged at 3000g for 10 minutes at 4°C. Supernatants containing sera were collected into new tubes and stored at -80°C until processing.

### Spleen sampling and preparation

Mice were anesthetized with isoflurane followed by final sacrifice using cervical dissociation. Spleen were harvested in FACS Buffer (PBS 1X, 3% FBS, 2mM EDTA) and digested using spleen dissociation kit and GentleMACS Octo Dissociator with Heaters (Miltenyi Biotec) per manufacturer’s instructions. Undigested tissues and debris were removed by filtering the cellular solution through a 70μM filter in FACS Buffer. Cell number was evaluated using a Luna-FL™ automated cell counter (Logos Biosystems).

### Flow cytometry

All cells were labeled with antibody cocktails and incubated for 30 min at 4°C in the dark. The antibodies and fluorescent reagents used in this study are listed in **Supplementary Table 6**. Cells were then washed in FACS Buffer before flow cytometry acquisition (Attune NxT, ThermoFisher). Data analysis was performed using FlowJo (BD Biosciences) and Prism (GraphPad) softwares. The gating strategy is shown in **Supplementary Figure 2**.

### Cytokine quantification by Multiplex ELISA

Human IL-6, CCL2 (MCP-1), G-CSF, IFN-α2, CCL5 (RANTES), IL-2, IFN-γ, IL-7, IL-1RA, CXCL8 (IL-8), TNF-α, CXCL10 (IP-10), CCL3 (MIP-1a), IL-10 were analyzed in serum using LEGENDPlex™ COVID-19 Cytokine Storm Panel 1 (BioLegend, #741089), following manufacturer’s instructions.

### ALAT, AST and SAA dosages

Frozen serum samples were thawed and Alanine Aminotransferase (ALAT), Aspartate aminotransferase (AST) and serum amyloid A (SAA) were measured. Assays were performed by Iodolab (Lyon, France). ALAT and AST were tested using a colorimetric assay from Diasys according to manufacturer’s instructions, and read on a Biomajesty 6010/c analyzer (Diasys). Quantification ranges for these 2 assays were from 0.6 to 1 200 U/L for ALAT and 1.2 to 1200 U/L for ASAT. Samples were tested undiluted. SAA was measured using an ELISA kit from LifeDiagnostic according to the manufacturer’s protocol and read using an ELISA Bioteck ELX800 reader. The quantification range was from 7.8 ng/mL to 2.5 10^6^ ng/mL. Samples were diluted from 1/100 to 1/5 000.

### Statistical analyses

Quantification and statistical analysis were performed using Excel and GraphPad Prism. The numerical data are presented as means ± SEM. The differences were determined by two-way ANOVA Multiple comparison and P value < 0.05 was considered statistically significant (*). **P<0.01, ***P<0.001, ****P<0.0001.

## Results

### hFlt3L pre-treatment enhances OKT3-induced cytokine release, body weight loss, and temperature drop in BRGSF-CBC mice

To investigate the potential use of BRGSF-CBC mice as a preclinical model for CRS assessment, and the crucial role of myeloid and dendritic cells in this process, mice were pre-treated or not with hFlt3L to boost human myeloid and dendritic compartments and injected with the known CRS-inducing OKT3 antibody or control isotype (**Supplementary Figure 1**).

**Figure 1:**
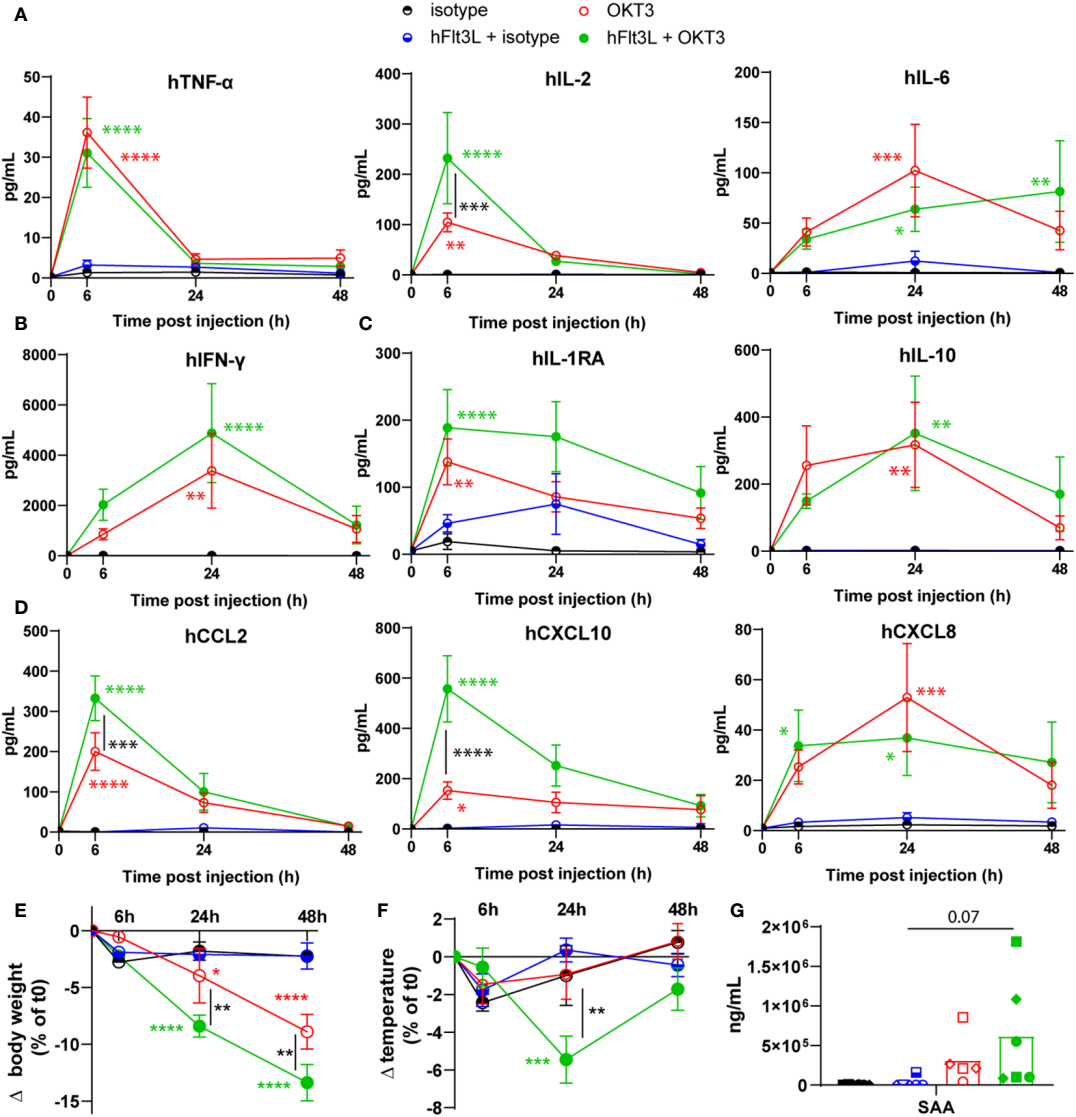
OKT3 treatment induces cytokine release, body weight loss, and temperature drop in BRGSF-CBC mice. Presence of human proinflammatory cytokines TNF-α, IL-2, and IL-6 **(A)**, Type II interferon IFN-ɣ **(B)**, regulatory cytokines IL1-RA and IL-10 **(C)**, and chemokines CCL2, CXCL10, and CXCL8 **(D)** in mice sera was tested at 6h, 24h, and 48h. Serum cytokine levels are shown as mean (line) and standard error (bars). Human, CCL5, G-CSF, IFN-α2, IL-7, and CCL3 were not detected in the samples. Body weight **(E)** and temperature **(F)** were measured in all mice at 6h, 24h, and 48h post-injection. Serum levels of Serum Amyloid A (SAA) was tested at 48h **(G)**. Individual donors are identified by symbol shapes, as indicated in **Supplementary Table 1**. Green and red stars correspond to statistical tests for the indicated group, at one time point compared to T0. Black stars correspond to statistical differences between hFlt3L treated and non-treated groups.

OKT3 treatment induced the significant release of human pro-inflammatory cytokines TNF-α, IL-2, and IL-6 (**Figure 1A**), human type II interferon, IFN-γ (**Figure 1B**), human regulatory cytokines IL-1RA and IL-10 (**Figure 1C**), and human chemokines CCL2, CXCL10 and CXCL8 (**Figure 1D**). Interestingly, hFlt3L pre-treatment increased the OKT3-dependent release of IL-2, CCL2, and CXCL10 (**Figures 1A, D**). Serum levels of TNF-α and IL-2, known markers of T cells activation, peaked 6h post OKT3 treatment, while myeloid and dendritic-associated cytokines IL-6, IL-10 and CXCL8 were increased in sera at later time points (24h and 48h). These results are consistent with a primary activation of T cells by CD3-targeting OKT3 treatment, followed by myeloid and dendritic cells activation, as recently proposed (18–20). To further evaluate the potential toxicity of OKT3 treatment in BRGSF-CBC mice, body weight and temperature were followed. A decrease in body weight was observed in OKT3-treated mice, and significantly enhanced in hFlt3L-boosted BRGSF-CBC mice (**Figure 1E**). A drop in temperature was observed at 24h post-OKT3 injection, only in hFlt3L pre-treated mice (**Figure 1F**). Additionally, serum amyloid A (SAA) presence in sera was increased in OKT3-treated mice (**Figure 1G**; p=0.07), suggesting acute inflammation. No changes were observed for Aspartate aminotransferase ASAT and Alanine aminotransferase ALAT (not shown), known markers of liver toxicity.

These data show that OKT3 treatment in BRGSF-CBC mice induces the release of human cytokines, body weight loss, and temperature drop, all associated with acute inflammation and CRS. In addition, they suggest that myeloid and dendritic cells, boosted by hFlt3L pre-treatment, enhance OKT3-induced features.

### OKT3’s impact on human immune cell distribution is intensified by hFlt3L boost in BRGSF-CBC mice

To identify the effect of OKT3 on immune cell distribution, spleens of all mice were collected 48h post-injection and analyzed by flow cytometry.

**Figure 2:**
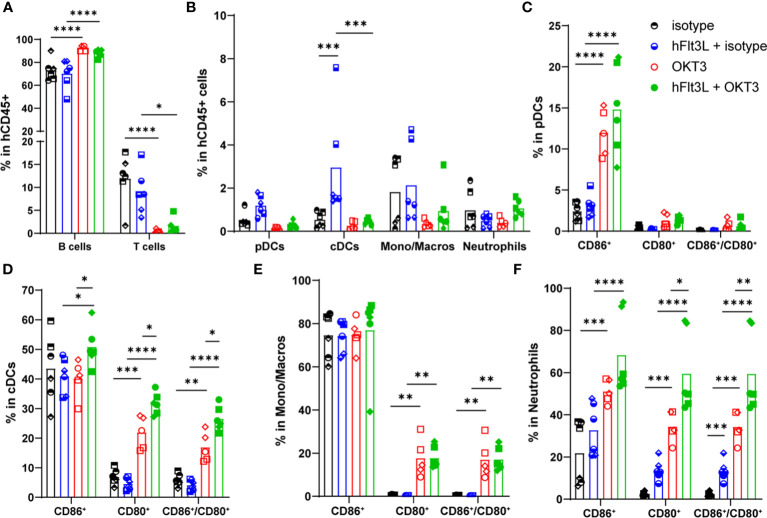
OKT3 treatment affects human immune cell distribution in BRGSF-CBC mice. Percentages of B (CD19^+^) and T (TCRb^+^) cells **(A)**, pDCs, cDCs, monocytes/macrophages, and neutrophils **(B)** in total human immune cells (CD45^+^) were analyzed from all mice spleens 48h after OKT3 treatment. Percentages of activated pDCs **(C)**, cDCs **(D)**, monocytes/macrophages **(E)**, and neutrophils **(F)** were determined as single CD86^+^, single CD80^+^, and double CD80^+^/CD86^+^ cells in each population. Individual donors are identified by symbol shapes, as indicated in **Supplementary Table 1**. Gating strategy is shown in **Supplementary Figure 2**. P value < 0.05 was considered statistically significant (*). **P<0.01, ***P<0.001, ****P<0.0001.

As described in other humanized models and in patients (21), OKT3 induced T cells’ decrease (**Figure 2A**; **Supplementary Figure 3A**). The absolute number of hCD45^+^ cells was slightly but significantly decreased upon OKT3 treatment in hFlt3L pre-treated mice only (**Supplementary Figure 3B**), but no significant impact was observed on the humanization rate (**Supplementary Figure 3C**). No effect was observed on neutrophils (**Figure 2B**; **Supplementary Figure 3D**) and NK cells percentage (not shown). Interestingly, pDCs, cDCs and monocytes/macrophages percentages and absolute numbers were depleted in OKT3-treated mice (**Figure 2B**; **Supplementary Figure 3D**). As expected, hFlt3L treatment alone increased proportions of cDC and pDC (8) (**Figure 2B**). Finally, OKT3’s effect on myeloid and dendritic cells was enhanced, and only significant, with hFlt3L pre-treatment (**Figure 2B**; **Supplementary Figure 3D**), demonstrating again that OKT3’s impact on myeloid and dendritic cells is best observed in hFlt3L-boosted BRGSF-CBC.

CD86, CD80 and HLA-DR markers were analyzed to evaluate myeloid and dendritic cells’ activation and maturation. In pDCs, OKT3 treatment induced an increase in CD86^+^ percentage (**Figure 2C**), together with upregulated HLA-DR and CD80 expression levels, although not significant for CD80 (**Supplementary Figure 3E**). Similarly, OKT3-treated mice showed increased percentages of CD86^+^, CD80^+^, and CD86^+^/CD80^+^ double positive cDCs (**Figure 2D**), while only a non-significant increase of CD80 expression level was observed (**Supplementary Figure 3F**). CD80^+^ and CD86^+^/CD80^+^ monocytes/macrophages percentages were significantly increased upon OKT3 treatment (**Figure 2E**), accompanied with CD80, CD86 and HLA-DR expression levels induction (**Supplementary Figure 3G**), although only significant for HLA-DR. Finally, percentages of CD86^+^, CD80^+^, and CD86^+^/CD80^+^ neutrophils, as well as CD80, CD86, and HLA-DR expression levels, were increased upon OKT3 injection (**Figure 2F**; **Supplementary Figure 3H**). For pDCs, cDCs, and neutrophils, OKT3-induced activation was enhanced by hFlt3L pre-treatment (**Figures 2C, D, F**; **Supplementary Figure 3H**), demonstrating that hFlt3L-treated BRGSF-CBC mice display enhanced OKT3’s induced features. Importantly, and as previously described (8), hFlt3L injections boosted myeloid and dendritic cells’ percentages (**Figure 2B**), and increased the total number of hCD45^+^ cells, with the strongest effect on dendritic cells (**Supplementary Figures 3B, D**), but did not induce major activation of these cells (**Figures 2C–F**; **Supplementary Figures 3E–H**), confirming that myeloid and dendritic cells activation is due to OKT3 treatment alone. Taken together, these data show that OKT3 treatment induces changes in human immune cell distribution (depletion of T, myeloid and dendritic cells), and myeloid and dendritic cells’ activation. Additionally, increasing the number and percentages of human myeloid and dendritic cells, through hFlt3L boost, amplifies OKT3-induced CRS features, suggesting a significant role of these cells in CRS development.

### Infliximab pre-treatment alleviates OKT3-induced CRS features in hFlt3L-boosted BRGSF-CBC mice

To test the efficacy of a clinical CRS-managing therapy in this model, BRGSF-CBC mice were treated with anti-TNF-α antibody Infliximab, before receiving OKT3 injections (**Supplementary Figure 4A**). Again, OKT3 treatment induced CRS-associated features, namely increased human cytokine serum levels, immune cell distribution and activation alteration, and a drop in body weight. Mice pre-treated with Infliximab showed reduced serum levels of IL-2, IL-6, IL-1RA, IL-10, CCL2, CXCL10, and CXCL8, compared to isotype pre-treated mice (**Supplementary Figure 4B**). Infliximab pre-treated mice also showed reduced serum levels of TNF-α and IFN-γ, compared to the OKT3 treated group (p<0.01). Control isotype pre-treatment impacted the release of these two cytokines. Interestingly, pDCs, cDCs, and monocytes/macrophages depletion seemed prevented by Infliximab pre-treatment (**Supplementary Figure 4C**), while T cells depletion and immune cells’ activation were not impacted (**Supplementary Figures 4D, E**). Lastly, OKT3-induced body weight loss was significantly attenuated by TNF-α inhibition (**Supplementary Figure 4F**). These data show that anti-TNF-α pre-treatment can hamper or lessen most effects of OKT3-induced CRS in hFlt3L-boosted BRGSF-CBC mice, thus providing a relevant preclinical model for CRS-managing treatments’ assessment.

### OKT3-induced CRS features are broadened in hFlt3L-boosted BRGSF-CBC compared to BRGSF-PBMC

As OKT3 treatment was previously shown to induce CRS features in PBMC-HIS mice, its effect was compared in BRGSF-PBMC and hFlt3L-boosted BRGSF-CBC mice (**Supplementary Figure 5A**).

**Figure 3:**
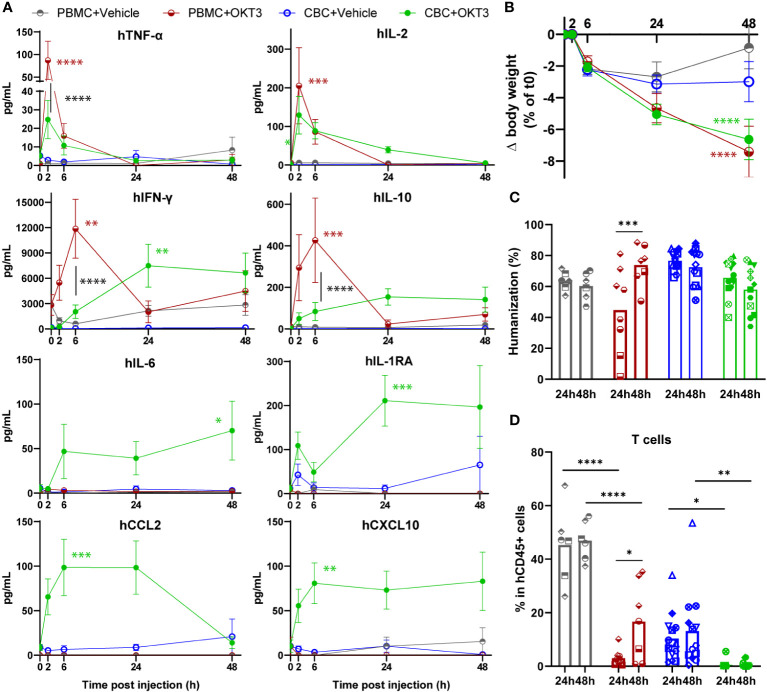
OKT3 treatment induces cytokine release, body weight loss and immune cell distribution alteration in BRGSF-PBMC and hFlt3L-boosted BRGSF-CBC mice. Presence of human TNF-α, IL-2, IFN-ɣ, IL-10, IL-6, IL1-RA, CCL2, and CXCL10 in mice sera was tested at 2h, 6h, 24h, and 48h **(A)**. Serum cytokine levels are shown as mean (line) and standard error (bars). Body weight was measured in all mice at 2h, 6h, 24h, and 48h post-injection **(B)**. Humanization rate in splenocytes of all Vehicle-injected mice were determined by flow cytometry **(C)**. T cells (TCRb^+^) in total human immune cells (CD45^+^) were analyzed from all mice spleens, 24h or 48h after OKT3 treatment **(D)**. Individual donors are identified by symbol shapes, as indicated in **Supplementary Table 3**. Green and red stars correspond to statistical tests for the indicated group, at one time point compared to T0. Black stars correspond to statistical differences between groups. P value < 0.05 was considered statistically significant (*). **P<0.01, ***P<0.001, ****P<0.0001.

Interestingly, these models showed specific differences before OKT3 injection. BRGSF-PBMC mice’s humanization rate seemed more variable than BRGSF-CBC’s, with 3 out of 7 PBMC donors and 1 out of 10 CBC donors giving mice with a humanization rate <30% (**Supplementary Figure 5B**). These mice were excluded from further analysis. Immune cell distribution was significantly different in these models, as BRGSF-PBMC mice immune system is mostly constituted of T and B cells, whereas BRGSF-CBC’s shows a lower percentage of T cells, higher percentage of B cells, and most importantly, detectable percentages of cDCs, monocytes/macrophages, and NK cells (**Supplementary Figure 5C**). In addition, 70% and 3% of T cells in BRGSF-PBMC mice were effector memory (EM) and naïve cells respectively, while 30% of T cells were naïve in BRGSF-CBC mice (**Supplementary Figure 5D**). Furthermore, T cells from BRGSF-PBMC mice exhibited high expression of CD25 and LAG3, confirming a pre-activation/exhaustion status of T cells in BRGSF-PBMC mice (**Supplementary Figure 5E**). Finally, serum levels of IFN-γ were higher in BRGSF-PBMC compared to BRGSF-CBC mice (**Figure 3A**, grey vs blue line). These data show that BRGSF-CBC mice display a more diversified immune system (B and T cells, myeloid, dendritic, and NK cells), while BRGSF-PBMC mice’s present mostly activated T and B cells at steady state.

Upon OKT3 treatment, human cytokine serum levels were increased with significant differences in the two models (**Figure 3A**). TNF-α and IL-2 were transiently detected 2h post OKT3 treatment in both models, at a significantly higher level in BRGSF-PBMC sera for TNF-α. IFN-γ and IL-10 serum levels were also increased upon OKT3 treatment, but showed different profiles with a peak at 6h for BRGSF-PBMC, and a maintained increase starting at 24h in BRGSF-CBC sera. These data are consistent with a primary activation of T cells upon anti-CD3 OKT3 treatment. Interestingly, serum levels of myeloid-associated cytokines IL-6, IL-1RA, CCL2, and CXCL10 were only increased upon OKT3 treatment in BRGSF-CBC mice, consistent with the presence of myeloid and dendritic cells in this model. Both models showed a significant body weight loss upon OKT3 injection (**Figure 3B**). Splenocytes analysis revealed a transient variability of the humanization rate of BRGSF-PBMC mice at 24h, consistent with a high percentage of OKT3-targeted T cells in these mice, that led to a lower humanization rate at 24h compared to 48h in this group (**Figure 3C**). As previously shown, T cells were depleted in both models at 24h and 48h (**Figure 3D**). Interestingly, while T cells depletion was complete and lasting in BRGSF-CBC mice (**Figure 3D**), T cells percentage was significantly higher at 48h compared to 24h in BRGSF-PBMC mice, suggesting a recovery from OKT3’s effect on immune cell distribution in this model, most likely due to the initial higher number of T cells in BRGSF-PBMC vs BRGSF-CBC mice. T cells depletion was accompanied by increased percentages of CD25^+^ and LAG3^+^ T cells, only significant at 24h (**Supplementary Figure 5F**), demonstrating that OKT3-induced T cell depletion is due to their activation and exhaustion, and suggesting again a transient impact of OKT3 in BRGSF-PBMC mice. T cell activation data for BRGSF-CBC mice could not be obtained as T cells were entirely depleted upon OKT3 treatment in this group at both studied time points. Finally, cDCs were depleted upon OKT3 injection in BRGSF-CBC mice at 24h and 48h (**Supplementary Figure 5G**), with an increased percentage of CD86^+^ and higher expression level of CD86 in cDCs (**Supplementary Figure 5H**), confirming the activation of remaining cDCs in this model. cDCs activation data for BRGSF-PBMC mice could not be obtained as cDCs are mostly absent in this model. Taken together, these data show that although both models are responsive to OKT3 treatment, hFlt3L-boosted BRGSF-CBC mice display a broader range of CRS features than BRGSF-PBMC, most likely due to their more varied human immune system. These data are consistent with our previous observation that myeloid and dendritic cells enhance OKT3-induced CRS-features.

### hFlt3L-boosted BRGSF-CBC mice show CRS-associated features upon treatment with CRS-inducing therapeutic antibodies

As hFlt3L-boosted BRGSF-CBC mice proved more responsive to OKT3 treatment than BRGSF-PBMC mice, their reliability to predict CRS was tested with other CRS-inducing compounds. Blinatumomab, an anti-hCD3/anti-hCD19 bispecific antibody approved for the treatment of Philadelphia chromosome-negative B-cell acute lymphoblastic leukemia patients, and known to induce CRS (15, 22, 23), was tested in hFlt3L-boosted BRGSF-CBC mice (**Supplementary Figure 6A**). A significant increase in serum levels of human TNF-α, IL-6, IFN-γ, CCL2, and CXCL10 was observed at early time points at 5 µg/kg (**Supplementary Figure 6B**). Serum levels of IL-2 and IL-10 were also increased, although not significantly. Interestingly, Blinatumomab treatment induced a decrease in body temperature that was transient at low concentration (2µg/kg), but sustained and significant at 5 µg/kg (**Supplementary Figure 6C**).

**Figure 4:**
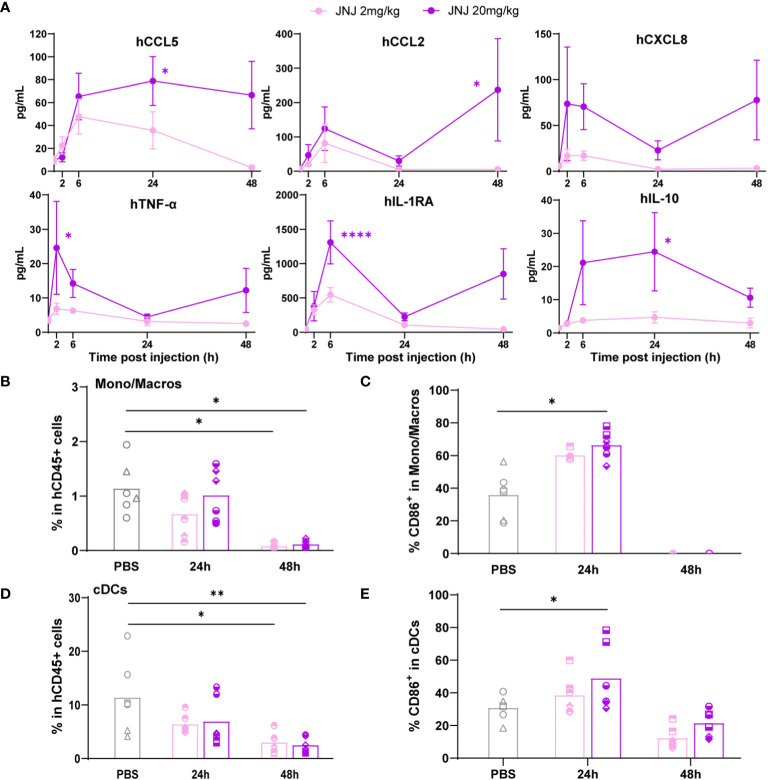
Anti-VISTA therapeutic antibody JNJ treatment induces cytokine release and myeloid cells depletion in hFlt3L-boosted BRGSF-CBC mice. Cytokine serum levels **(A)** were analyzed in all mice upon JNJ treatment at indicated time points. Percentages of monocytes/macrophages in total human immune cells **(B)**, CD86^+^ activated monocytes/macrophages **(C)**, cDCs in total human immune cells **(D)**, and CD86^+^ activated cDCs **(E)** were analyzed from mice spleens at 24h and 48h by flow cytometry. Individual donors are identified by symbol shapes, as indicated in **Supplementary Table 5**. Purple stars correspond to statistical tests for the indicated group, at one time point compared to T0. Black stars correspond to statistical differences between groups. P value < 0.05 was considered statistically significant (*). **P<0.01, ***P<0.001, ****P<0.0001.

Last, hFlt3L-boosted BRGSF-CBC mice were injected with a myeloid-targeting anti-hVISTA antibody, JNJ (**Supplementary Figure 7A**), used in a phase I clinical trial run by Janssen in patients with advanced solid tumors in 2016 and terminated after the occurrence of CRS-related side effects (16, 24). Interestingly, JNJ-induced cytokine release was dose-dependent, and included preferentially monocytes/macrophages secreted human chemokines such as CCL5, CCL2, CXCL8, and CXCL10 (**Figure 4A**; **Supplementary Figure 7B**). TNF-α, IL-1RA, and IL-10 serum levels were also increased. Splenocytes flow cytometry analysis showed a depletion of monocytes/macrophages percentage at 48h (**Figure 4B**), preceded at 24h by a significant increase in the percentage of CD86^+^ activated monocytes/macrophages (**Figure 4C**). Similarly, cDCs percentage was decreased at 48h (**Figure 4D**), while the percentage of activated CD86^+^ cDCs was increased at 24h upon high-dose treatment (**Figure 4E**). These changes were not observed in pDCs, most likely due to a lower expression of VISTA on this population (**Supplementary Figures 7C–E**). These data show that hFlt3L-boosted BRGSF-CBC mice are responsive to a variety of known CRS-inducing agents, and confirm that they represent a good model to test and predict CRS-like toxicity in preclinical settings, for compounds targeting the lymphoid, myeloid, or dendritic compartments.

## Discussion

Humanized mice are increasingly used to test immunotherapies, and the pros and cons of these models have been extensively discussed (25). As the human immune system in PBMC-HIS mice is composed of mature adult immune cells specific of the donor’s immune history, it can greatly impact, and bias, immune responses. In addition, immune cells in this model are mostly constituted of mature T cells, as observed here in BRGSF-PBMC mice, making PBMC-HIS mice more prone to develop acute Graft versus Host Disease (GvHD). CBC-HIS mice do not develop acute GvHD and their immune system is composed of more naïve cells, but the distribution of immune cells in this model is different from an adult human immune system, as implanted cells recreate immune populations in a murine environment. These models are useful for different applications, but knowing their limitations is essential to properly design experiments and interpret data (7, 25).

As previously shown, BRGSF-CBC mice display significant presence of human myeloid and dendritic cells that can be boosted upon hFlt3L injections (8, 9). Here, BRGSF-CBC mice were used to test their response to known CRS-inducing compounds. We showed that hallmark cytokines of CRS such as human IFN-γ and TNF-α were induced upon anti-CD3 antibody OKT3 treatment. This CRS-inducing treatment also led to T, myeloid, and dendritic cells depletion, and activation of remnant human myeloid and dendritic cells. These changes were accompanied with a loss of body weight and a temperature drop. Interestingly, CBC-reconstituted NSG-HIS mice were previously described as unresponsive to a 6h-OKT3 treatment (6), although serum levels of IL-2, IL-10, TNF-α, and IFN-γ were significantly increased upon OKT3 injection. Here we showed that hFlt3L-boosted BRGSF-CBC mice are responsive to this treatment, with increased cytokine serum levels starting at 6h and T, myeloid, and dendritic cells depletion. A significant decrease of body weight and temperature starting at 24h, was also observed, suggesting that boosted myeloid and dendritic compartments allow for amplified CRS features upon OKT3 treatment in BRGSF-CBC mice. Recent studies propose that T-cell engagers-induced CRS could start with the release of TNF-α by activated T-cells, which would then activate myeloid cells and amplify cytokine release (18–20). This is consistent with our observations in hFlt3L-boosted BRGSF-CBC where therapeutics-induced release of cytokines by activated T cells induces myeloid and dendritic cells’ activation, illustrated by the expression of CD86 and CD80, leading to myeloid and dendritic cells’ exhaustion and thus depletion at 24h and 48h.

We also demonstrated that anti-TNF-α Infliximab treatment, a clinical CRS-managing therapy, lessened OKT3-induced CRS features in hFlt3L-boosted BRGSF-CBC mice. Cytokine serum levels were reduced, myeloid and dendritic cells depletion was reversed, and body weight loss was attenuated by anti-TNF-α injection, whereas OKT3-induced T cells depletion, myeloid and dendritic cells activation, and temperature drop were not prevented. This is in accordance with the previous observation that TNF-α secreted by activated T-cells is responsible for myeloid cells activation and amplification of CRS features (18–20). These data thus suggest that BRGSF-CBC mice represent a valuable model for the assessment of CRS-managing therapies and confirm their relevance for the study of CRS mechanisms and human myeloid and dendritic cells contribution.

In addition, we showed that BRGSF-PBMC mice could not display myeloid and dendritic cells depletion or activation, as they exhibit very low levels of these cells, and released fewer cytokines than BRGSF-CBC mice upon OKT3 treatment. These results strongly support a role for human myeloid and dendritic cells in CRS development, as hFlt3L-boosted BRGSF-CBC mice developed a wider range of CRS-like features. As Flk2 is invalidated in BRGSF mice, hFlt3L cannot directly act on mouse mature and progenitor DC. Nevertheless, secreted human cytokines may cross-react on bystander murine myeloid and dendritic cells and thus a contribution of these cells to the induction of CRS cannot be excluded. Further investigations need to be performed to decipher the respective roles of murine and human cells in CRS induction in this model.

Finally, we show that hFlt3L-boosted BRGSF-CBC are responsive to other known CRS-inducing agents (15, 16), targeting not only T, but also B, myeloid, and dendritic cells. Indeed, treatment with anti-CD19/anti-CD3 bispecific antibody Blinatumomab induced the release of hallmark cytokines of CRS, as well as a temperature drop. Myeloid-targeting with anti-hVISTA antibody JNJ induced the release of CRS-associated human cytokines, particularly monocytes/macrophages secreted and chemo-attractants’ chemokines such as CCL2, CCL5, and CXCL8. Additionally, JNJ treatment led to a significant activation of monocytes/macrophages and cDCs, followed by a depletion of these cells’ percentages, most likely due to activation-induced cell death or FcɣR-mediated depletion. Interestingly, CRS features induced by Blinatumomab and JNJ administration differ from those observed after OKT3 treatment in nature and/or timing, suggesting that different time points should be tested when assessing CRS with a novel compound in this model. Importantly, hFlt3L-boosted BRGSF-CBC mice were used to assess the potential toxicity of a novel conditionally active anti-VISTA antibody (SNS-101), and showed no significant CRS features in preclinical settings (manuscript in preparation), as opposed to the anti-VISTA antibody JNJ used in this study. SNS-101 is now being evaluated in patients with advanced solid tumors in a phase I/II clinical trial (26). This thus validate the model’s relevancy for the preclinical assessment and translatability prediction of therapeutics-induced cytokine release.

Taken together, these data show that BRGSF-CBC mice exhibit a functional human immune system including lymphoid, myeloid, and dendritic compartments, and represent a reliable model to dissect the contribution of any of these compartments to CRS and assess CRS-managing therapies. The significant presence of human myeloid and dendritic cells in these mice enhances CRS-associated features and provides increased versatility for relevant testing of myeloid- or dendritic-targeting compounds.

## Data availability statement

The original contributions presented in the study are included in the article/**Supplementary Material**. Further inquiries can be directed to the corresponding authors.

## Ethics statement

Ethical approval was not required for the studies on humans in accordance with the local legislation and institutional requirements because only commercially available established cell lines were used. The animal study was approved by comité éthique VetAgro - Sup n°18. The study was conducted in accordance with the local legislation and institutional requirements.

## Author contributions

GM: Conceptualization, Data curation, Formal Analysis, Methodology, Validation, Writing – original draft, Writing – review & editing. AG: Formal Analysis, Investigation, Writing – review & editing. PM-J: Conceptualization, Data curation, Formal Analysis, Methodology, Validation, Writing – review & editing. FR-D: Conceptualization, Data curation, Formal Analysis, Methodology, Writing – original draft, Writing – review & editing. ZB: Methodology, Resources, Writing – review & editing. AC: Methodology, Resources, Writing – review & editing. AM: Methodology, Resources, Writing – review & editing. TT: Methodology, Resources, Writing – review & editing. AD: Methodology, Resources, Writing – review & editing. DC: Methodology, Resources, Writing – review & editing. LB: Methodology, Resources, Writing – review & editing. EvdH: Methodology, Resources, Writing – review & editing. AR: Data curation, Formal Analysis, Validation, Visualization, Writing – original draft, Writing – review & editing. KT: Conceptualization, Methodology, Supervision, Writing – review & editing.
