# Supplementary material for: Myeloid and dendritic cells enhance therapeutics-induced cytokine release syndrome features in humanized BRGSF-HIS preclinical model
Source: Front Immunol. 2024 Feb 7;15:1357716. doi: 10.3389/fimmu.2024.1357716 (PMC10880010; doi:10.3389/fimmu.2024.1357716)
Supplement: Supplementary file 1 [file DataSheet_1.docx]

Supplementary Material

### Supplementary Table 1: Mice identification and immunoprofiling at randomization (12 weeks) for OKT3 treatment of BRGSF-CBC mice with and without hFlt3L pre-treatment experiment

### Supplementary Table 2: Mice identification and immunoprofiling at randomization (12 weeks) for Infliximab rescue experiment in hFlt3L-boosted BRGSF-CBC mice

### Supplementary Table 3: Mice identification at randomization for OKT3 treatment of BRGSF-CBC and BRGSF-PBMC experiment, and immunoprofiling (at 12 weeks) for BRGSF-CBC mice included in this experiment

### Supplementary Table 4: Mice identification and immunoprofiling at randomization (12 weeks) for Blinatumomab experiment in hFlt3L-boosted BRGSF-CBC mice

### Supplementary Table 5: Mice identification and immunoprofiling at randomization (12 weeks) for anti-VISTA JNJ experiment in hFlt3L-boosted BRGSF-CBC mice

### Supplementary Table 6: List of reagents used for flow cytometry labeling

| **Target** | **Clone #** | **Provider** |
| --- | --- | --- |
|  |  |  |
| FcR Blocking Reagent |  | Miltenyi |
| LIVE/DEAD™ Fixable Aqua | L34957 | ThermoFisher |
| Anti-mouse CD45 | REA737 | Miltenyi |
| Anti-human CD45 | HI30 | Biolegend |
| Anti-human CD3 | UCHT1 | BD Biosciences |
| Anti-human CD3 | REA613 | Miltenyi |
| Anti-human TCRb | IP26 | BioLegend |
| Anti-human CD19 | REA675 | Miltenyi |
| Anti-human CD19 | HIB19 | BioLegend |
| Anti-human CD56 | REA196 | Miltenyi |
| Anti-human CD56 | HCD56 | BioLegend |
| Anti-human CD11c | Bu15 | BioLegend |
| Anti-human HLA-DR | L243 | BioLegend |
| Anti-human HLA-DR | LN3 | BioLegend |
| Anti-human CD14 | M5E2 | BD Biosciences |
| Anti-human CD16 | 3G8 | BioLegend |
| Anti-human CD16 | eBIOBC16 | ThermoFisher |
| Anti-human CD303 | REA693 | Miltenyi |
| Anti-human CD86 | BU63 | BioLegend |
| Anti-human CD86 | REA968 | Miltenyi |
| Anti-human CD80 | 2D10 | BioLegend |
| Anti-human CD123 | 6H6 | BioLegend |
| Anti-human CD45RA | HI100 | BioLegend |
| Anti-human CD197 (CCR7) | G043H47 | BioLegend |
| Anti-human CD25 | BC96 | BioLegend |
| Anti-human CD223 (LAG3) | 11C3C65 | BioLegend |
| Anti-human VISTA | 730804 | R&D systems |
| Anti-human VISTA | JNJ-61610588 | Provided by Sensei Biotherapeutics |

###
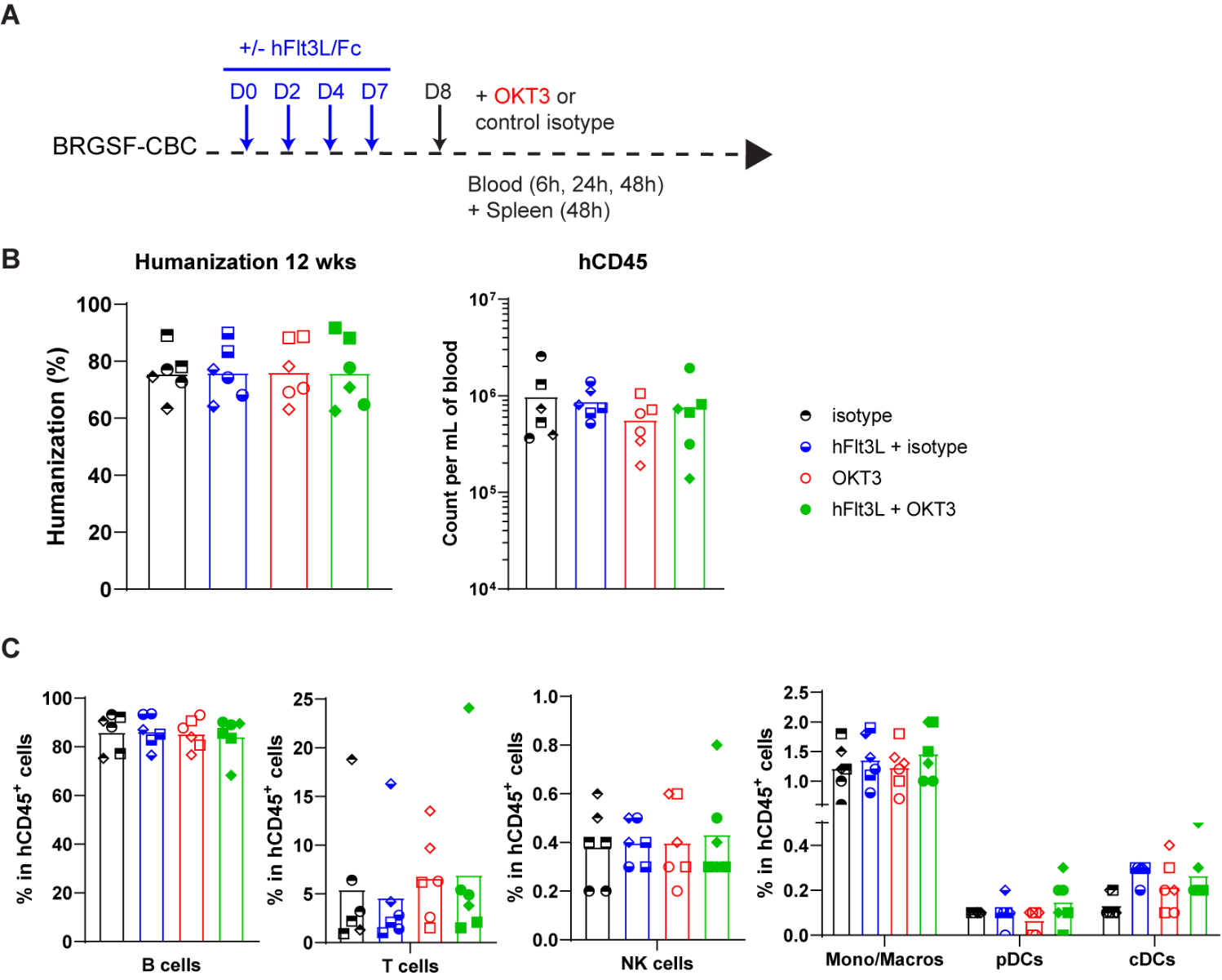
Supplementary Figure 1:

**Supplementary Figure 1.** Schematic of BRGSF-CBC mice treatments (A). At 21-22 weeks of age, hFlt3L pre-treated BRGSF-CBC mice received four injections of hFlt3L. All mice were injected by intravenous route at D8 with anti-CD3 OKT3, or mIgG2a. Blood was collected 6h, 24h, and 48h after treatment. Spleens were collected at sacrifice at 48h. Immunoprofiling at randomization (12 weeks) for all mice included in this experiment was performed by flow cytometry (B-C). Randomization groups and immunoprofiling at 12 weeks are detailed in Sup Table 1. Individual donors are identified by symbol shapes, as indicated in Sup Table 1. Gating strategy is shown in Sup Figure 2.


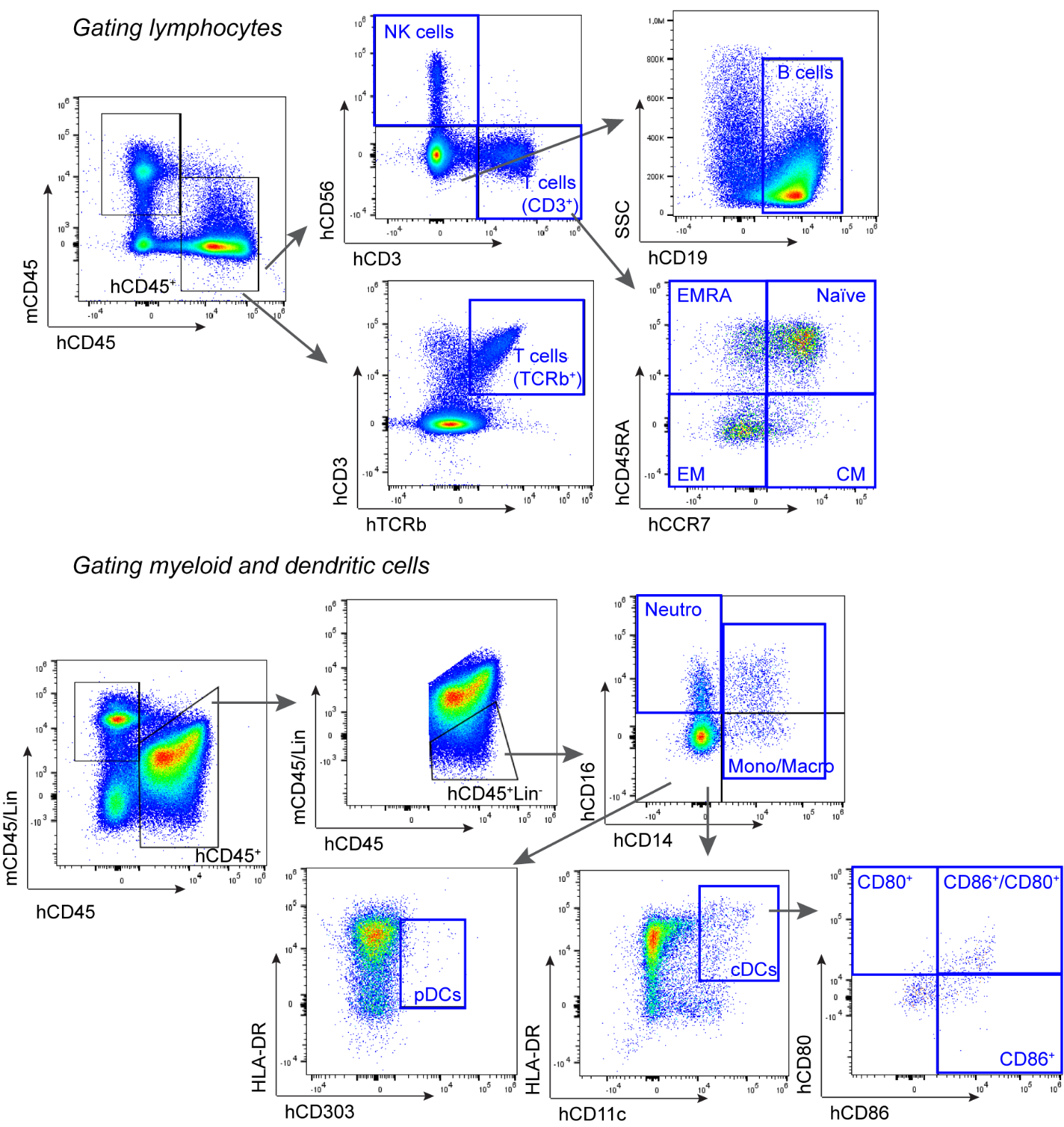
**Supplementary Figure 2**

**Supplementary Figure 2.** Representative gating strategy for flow cytometry analysis. EM: Effector Memory; EMRA: Effector Memory RA^+^; CM: Central Memory. Lin=hCD3/hCD56/hCD19. Neutro=Neutrophils. Mono/macro=Monocytes/macrophages. cDCs= conventional dendritic cells. pDCs=plasmacytoid dendritic cells. CD80^+^= single CD80 positive (CD86 negative). CD86^+^= single CD86 positive (CD80 negative).


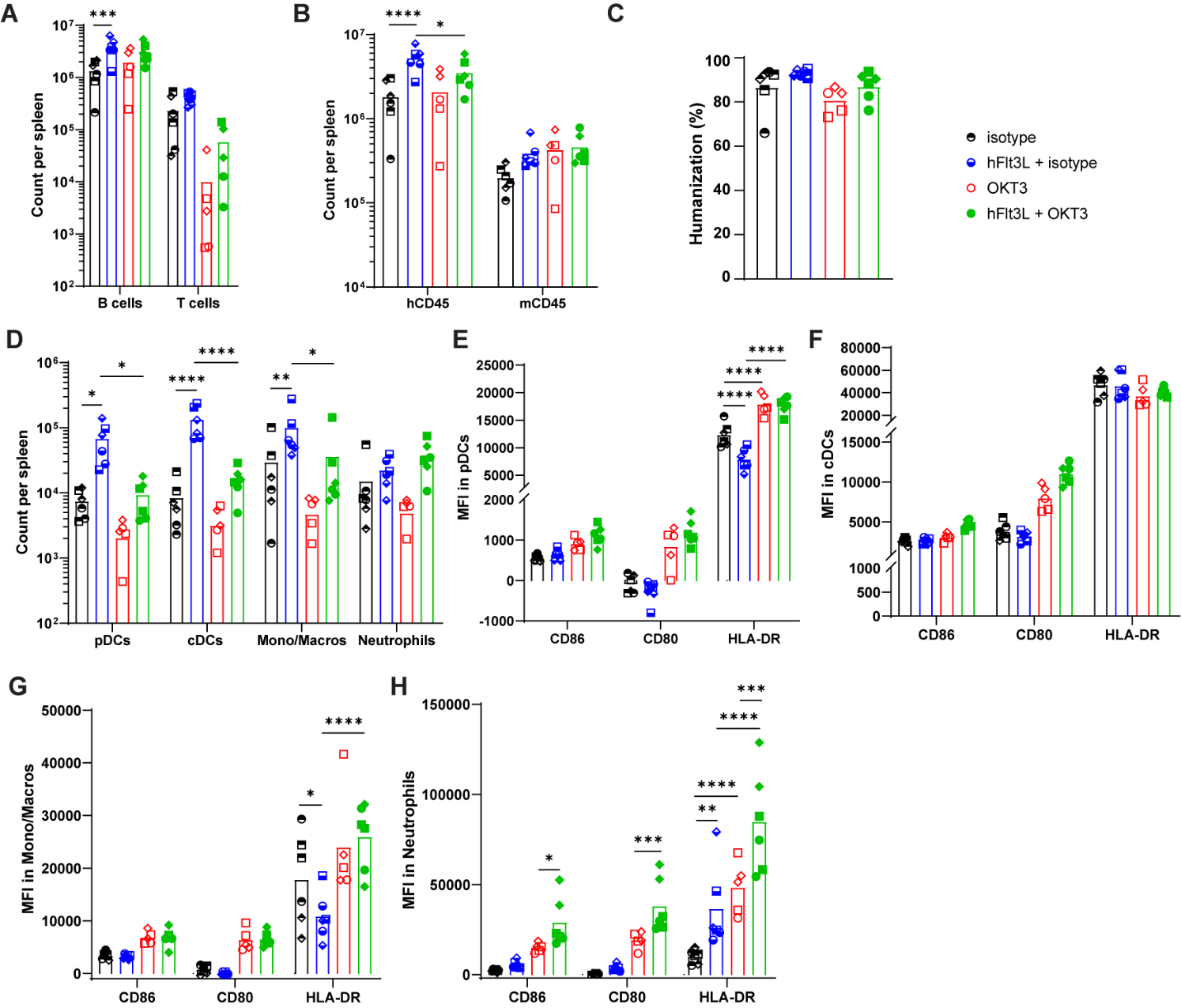
**Supplementary Figure 3**

**Supplementary Figure 3.** OKT treatment of BRGSF-CBC mice with or without hFlt3L injections. B and T (TCRb^+^) cells counts (A), hCD45^+^ and mCD45^+^ cell counts (B), humanization rate (C), and myeloid and dendritic populations cell counts (D) in the spleen at 48h were determined by flow cytometry. Expression levels of activation markers CD80, CD86, and HLA-DR, as mean fluorescence intensity (MFI), in pDCs (E), cDCs (F), Monocytes/Macrophages (G), and Neutrophils (H) were analyzed. Individual donors are identified by symbol shapes, as indicated in Sup Table 1. Gating strategy is shown in Sup Figure 2.

###
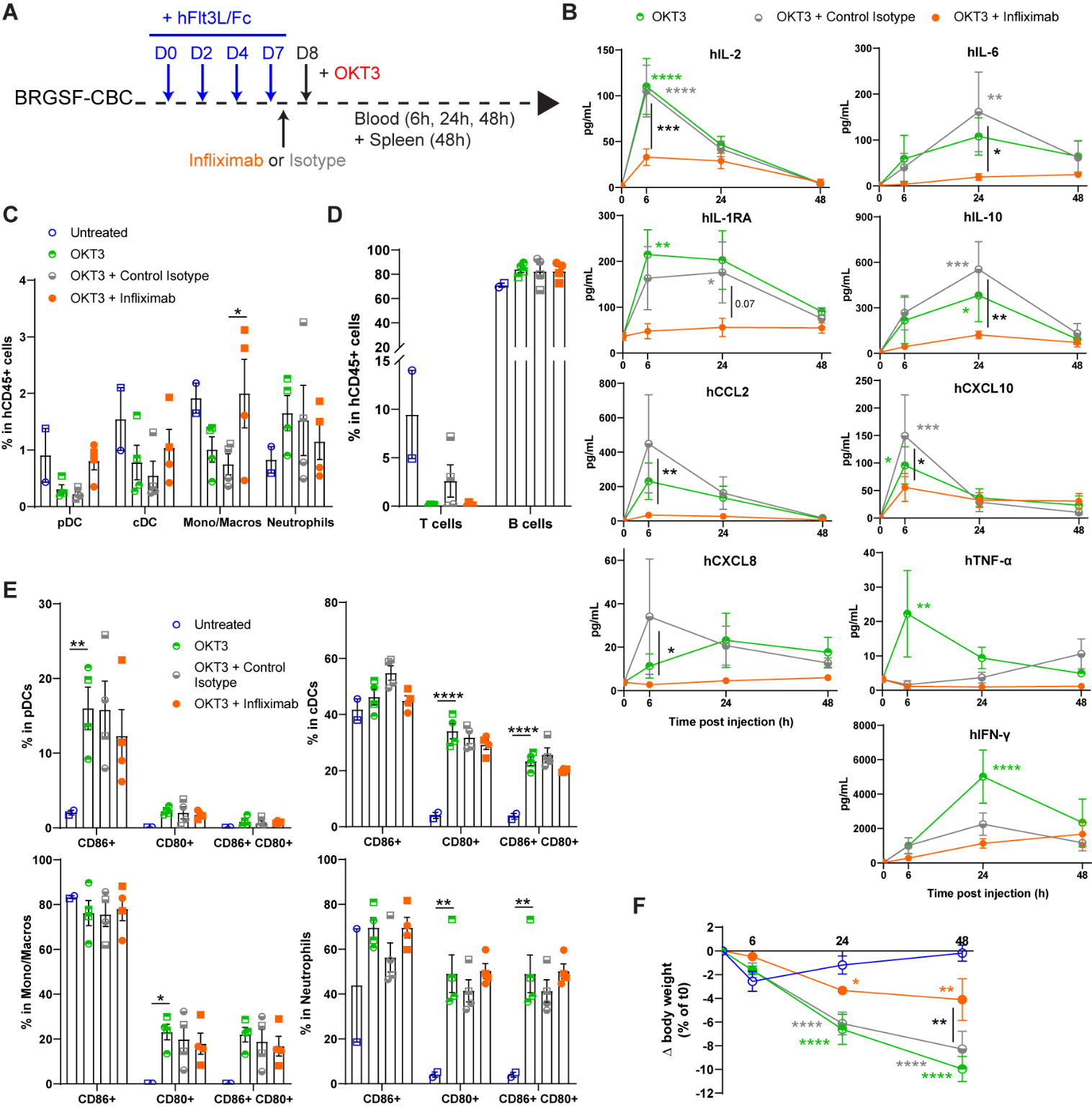
Supplementary Figure 4:

**Supplementary Figure 4.** Schematic of hFlt3L-boosted BRGSF-CBC mice Infliximab rescue treatment (A). At 21-22 weeks of age, all mice received four injections of hFlt3L. Mice were injected by intravenous route with Infliximab or control Isotype, one hour before receiving anti-CD3 OKT3 injection at D8. Blood was collected 6h, 24h, and 48h after OKT3 treatment. Spleens were collected at sacrifice at 48h. Serum levels of human cytokines were measured at indicated time points (B). Percentages of pDCs, cDCs, monocytes/macrophages, and neutrophils (C), and T (TCRb^+^) and B cells (D) in total human immune cells in the spleen at 48h were analyzed by flow cytometry. Percentages of activated pDCs, cDCs, monocytes/macrophages and neutrophils in the spleen at 48h were analyzed (E). Body weight was followed at 6h, 24h, and 48h after OKT3 treatment (F). Green, orange, and grey stars correspond to statistical tests for the indicated group, at one time point compared to T0. Randomization groups and immunoprofiling at 12 weeks are detailed in Sup Table 2. Individual donors are identified by symbol shapes, as indicated in Sup Table 2. Gating strategy is shown in Sup Figure 2.


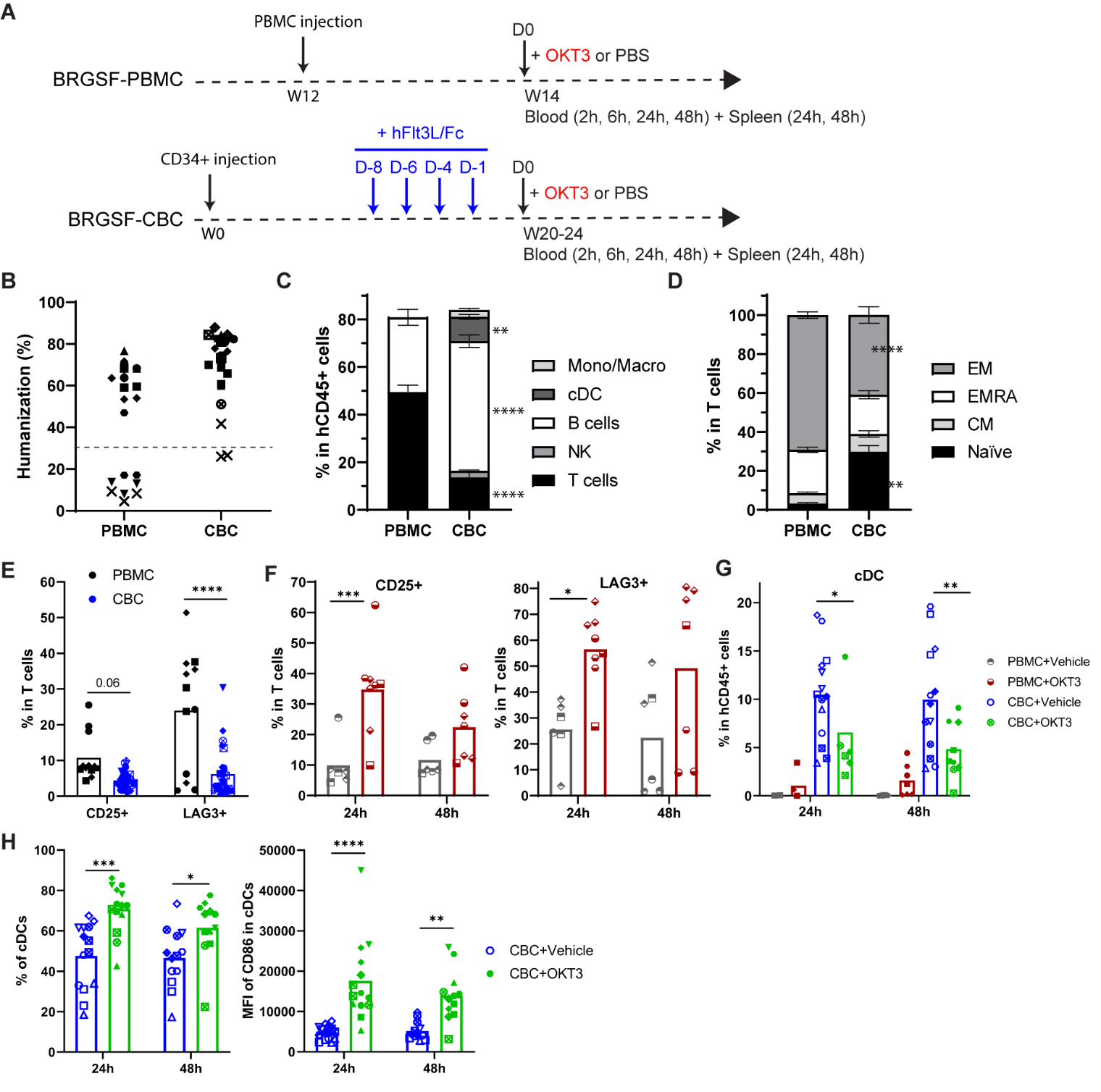
**Supplementary Figure 5:**

**Supplementary Figure 5**. Schematic of BRGSF-PBMC and hFlt3L-boosted BRGSF-CBC mice treatments (A, top panel). At 14 weeks of age, BRGSF-PBMC mice were injected by intravenous route at D0 with anti-CD3 OKT3, or vehicle (PBS). 19 to 23 weeks-old BRGSF-CBC mice received four injections of hFlt3L, followed a day later by an injection by intravenous route (D0) with anti-CD3 OKT3, or vehicle (PBS; A lower panel). Blood was collected 2h, 6h, 24h, and 48h after OKT3 treatment. Spleens were collected at sacrifice at 24h or 48h, and analyzed by flow cytometry. Humanization rate of all mice injected with vehicle (24h and 48h) were determined for all injected donors (B). Data from all vehicle-injected mice from donors with a humanization rate > 30% were analyzed to determine immune cell distribution (C), subpopulations of T cells (D), and percentages of CD25^+^ and LAG3^+^ T cells (E). Percentages of CD25^+^ (left panel) and LAG3^+^ (right panel) T cells in splenocytes of vehicle- and OKT3-treated BRGSF-PBMC mice were determined (F). Data for BRGSF-CBC mice could not be obtained as T cells are entirely depleted upon OKT3 treatment in this group. Percentages of cDCs in total human immune cells (CD45^+^) were analyzed from all mice spleens, 24h and 48h after OKT3 treatment (G). Percentages of activated CD86^+^ cDCs (left panel) and expression level of activation marker CD86 (right panel; as mean fluorescence intensity, MFI) were determined (H) for BRGSF-CBC groups. Data for BRGSF-PBMC mice could not be obtained as cDCs are absent in this model. Individual donors are identified by symbol shapes. Gating strategy is shown in Sup Figure 2. Randomization groups for all mice and immunoprofiling at 12 weeks for BRGSF-CBC mice are detailed in Sup Table 3. EM: Effector Memory; EMRA: Effector Memory RA^+^; CM: Central Memory.

### Supplementary Figure 6:


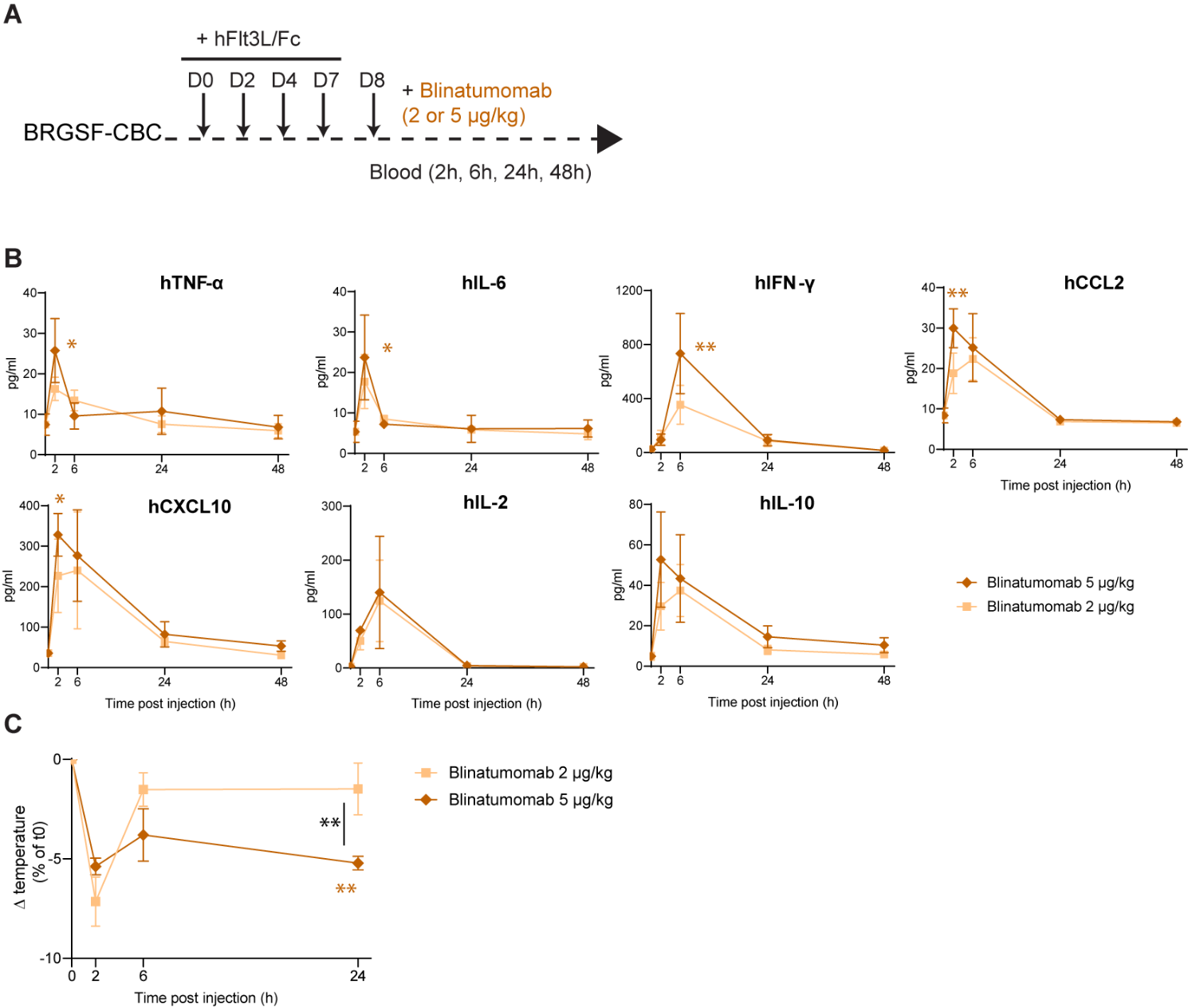


**Supplementary Figure 6.** Schematic of hFlt3L-boosted BRGSF-CBC mice Blinatumomab treatment (A). At 21-22 weeks of age, all mice received four injections of hFlt3L. Mice were injected by intravenous route with Blinatumomab at D8. Blood was collected 6h, 24h, and 48h after treatment. Spleens were collected at sacrifice at 48h. Cytokines serum levels were tested at 6h, 24h, and 48h (B). Body temperature was measured at 6h, 24h, and 48h after treatment (C). Brown stars correspond to statistical tests for the indicated group, at one time point compared to T0. Randomization groups and immunoprofiling at 12 weeks are detailed in Sup Table 4.

###
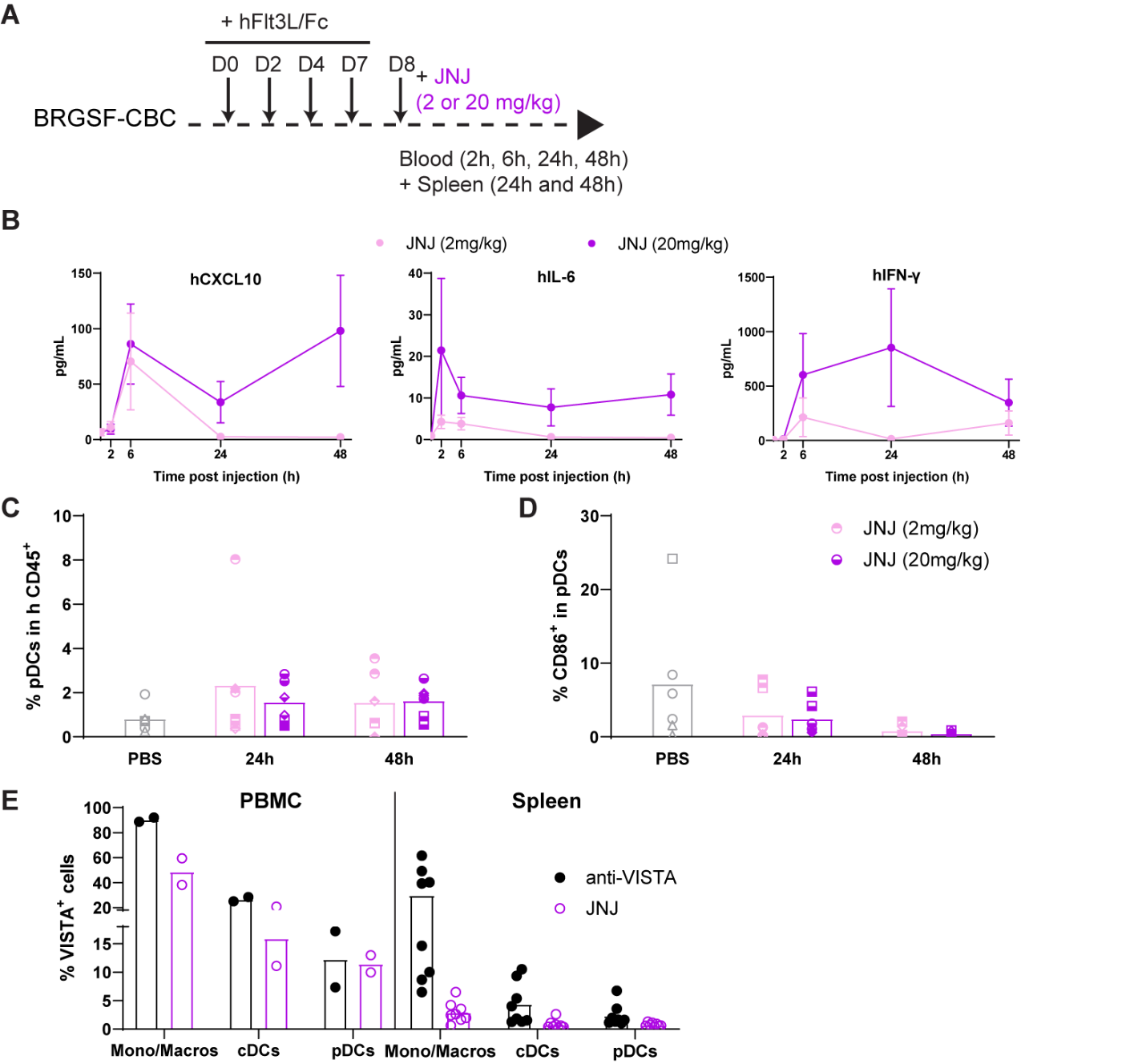
Supplementary Figure 7:

**Supplementary Figure 7.** Schematic of hFlt3L-boosted BRGSF-CBC mice anti-VISTA JNJ treatment (A). At 21-22 weeks of age, all mice received four injections of hFlt3L. Mice were injected by intravenous route with anti-VISTA JNJ at D8. Blood was collected 2h, 6h, 24h, and 48h after treatment. Spleens were collected at sacrifice at 24h or 48h. Cytokines serum levels were tested at 2h, 6h, 24h, and 48h (B). Percentages of pDCs in total human immune cells (C), and CD86^+^ cells in pDCs (D), were analyzed. Percentages of human VISTA^+^ monocytes/macrophages, cDCs, and pDCs in human PBMCs (E, left panel) and hFlt3L pre-treated BRGSF-CBC mice spleens (E, right panel), were assessed by flow cytometry using anti-human VISTA (clone 730804) or JNJ antibody. Randomization groups and immunoprofiling at 12 weeks are detailed in Sup Table 5. Individual donors are identified by symbol shapes, as indicated in Sup Table 5.
